# Loss of heterozygosity (LOH), malignancy grade and clonality in microdissected prostate cancer

**DOI:** 10.1038/sj.bjc.6690087

**Published:** 1999-02

**Authors:** A Hügel, N Wernert

**Affiliations:** Institute of Pathology, University of Bonn, PO Box 2120, Bonn, 53011, Germany

**Keywords:** prostate carcinoma, loss of heterozygosity

## Abstract

The aim of the present study was to find out whether increasing malignancy of prostate carcinoma correlates with an overall increase of loss of heterozygosity (LOH), and whether LOH typing of microdissected tumour areas can help to distinguish between multifocal or clonal tumour development. In 47 carcinomas analysed at 25 chromosomal loci, the overall LOH rate was found to be significantly lower in grade 1 areas (2.2%) compared with grade 2 (9.4%) and grade 3 areas (8.3%, *P* = 0.007). A similar tendency was found for the mean fractional allele loss (FAL, 0.043 for grade 1, 0.2 for grade 2 and 0.23 for grade 3, *P* = 0.0004). Of 20 tumours (65%) with LOH in several microdissected areas, 13 had identical losses at 1–4 loci within two or three areas, suggesting clonal development of these areas. Markers near *RB*, *DCC*, *BBC1*, *TP53* and at D13S325 (13q21–22) showed higher loss rates in grades 2 and 3 (between 25% and 44.4%) compared with grade 1 (0–6.6%). Tumour-suppressor genes (TSGs) near these loci might, thus, be important for tumour progression. *TP53* mutations were detected in 27%, but *BBC1* mutations in only 7%, of samples with LOH. Evaluation of all 25 loci in every tumour made evident that each prostate cancer has its own pattern of allelic losses. © 1999 Cancer Research Campaign


*British Journal of Cancer* (1999) **79**(3/4), 551–557
© 1999 Cancer Research Campaign
Article no. bjoc.1998.0087

### Loss of heterozygosity (LOH), malignancy grade and clonality in microdissected prostate cancer

**A Hügel and N Wernert**

Institute of Pathology, University of Bonn, PO Box 2120, 53011 Bonn, Germany

**Summary** The aim of the present study was to find out whether increasing malignancy of prostate carcinoma correlates with an overall increase of loss of heterozygosity (LOH), and whether LOH typing of microdissected tumour areas can help to distinguish between multifocal or clonal tumour development. In 47 carcinomas analysed at 25 chromosomal loci, the overall LOH rate was found to be significantly lower in grade 1 areas (2.2%) compared with grade 2 (9.4%) and grade 3 areas (8.3%, $P$ = 0.007). A similar tendency was found for the mean fractional allele loss (FAL, 0.043 for grade 1, 0.2 for grade 2 and 0.23 for grade 3, $P$ = 0.0004). Of 20 tumours (65%) with LOH in several microdissected areas, 13 had identical losses at 1–4 loci within two or three areas, suggesting clonal development of these areas. Markers near *RB*, *DCC*, *BBC1*, *TP53* and at D13S325 (13q21–22) showed higher loss rates in grades 2 and 3 (between 25% and 44.4%) compared with grade 1 (0–6.6%). Tumour-suppressor genes (TSGs) near these loci might, thus, be important for tumour progression. *TP53* mutations were detected in 27%, but *BBC1* mutations in only 7%, of samples with LOH. Evaluation of all 25 loci in every tumour made evident that each prostate cancer has its own pattern of allelic losses.

**Keywords:** prostate carcinoma; loss of heterozygosity

The clinical outcome of prostate cancer is strongly related to its differentiation and malignancy grade. Although well-differentiated tumours do not significantly affect patients' survival, less-differentiated neoplasms have a major impact on prognosis (Hanash et al, 1972; Böcking et al, 1982; Dhom, 1991). A significant proportion of prostate carcinomas are heterogeneous and pluriform neoplasms, which consist of several histological patterns with different biological properties (Dhom, 1991). Whether these patterns result from mulifocal or clonal tumour development is still poorly understood.

Like other neoplasms, prostate carcinoma is probably the result of a multistep carcinogenesis (Sandberg, 1992; Gao et al, 1995*a*). An overexpression of c-*ras*, c-*myc* and c-*sis* oncogenes has been reported (Fleming et al, 1986; Viola et al, 1986; Buttyan et al, 1987), but *ras* mutations are rare (Gumerlock et al, 1991; Moul et al, 1992). A role of *HER-2/neu* is not certain (Kuhn et al, 1993; Sadasivan et al, 1993). Recent evidence suggests that tumour-suppressor genes (TSGs) might be more important for the development of prostate carcinoma (Bookstein, 1994; Isaacs, 1995). Mutations and allelic losses (loss of heterozygosity or LOH) have been demonstrated for TSGs such as *TP53* (Gao et al, 1995*a*), *DCC* (deleted in colon carcinoma) (Gao et al, 1993), *APC* (adenomatous polyposis coli), *MCC* (mutated in colorectal cancer) (Gao et al, 1995*b*), *E-cadherin* (Umbas et al, 1992) and *BRCA1* (breast carcinoma-associated gene) (Gao et al, 1995*c*).

Few LOH studies have systematically analysed the relationship between malignancy grade or clinical stage and the frequency of allelic losses (Isaacs and Carter, 1991; Trapman et al, 1994; Macoska et al, 1995; Cunningham et al, 1996; Latil et al, 1996; Vocke et al, 1996), or taken into account intratumoral heterogeneity of prostate carcinoma (Konishi et al, 1995; Mirchandani et al, 1995). There is only one report of a significant increase of LOH at a single chromosomal locus (8p12–21) in advanced tumour stages (Trapman et al, 1994).

The aim of the present work was to evaluate whether increasing malignancy of prostate carcinoma goes along with a general increase in the frequency of LOH and the number of chromosomal loci concerned, and whether LOH typing can help to distinguish between multifocal or clonal tumour development. We analysed 47 carcinomas for LOH at 25 chromosomal loci near known or putative TSGs, evaluating 1–7 areas per tumour by microdissection. Two TSGs, *TP53* and *BBC1* (the breast basic conserved gene) (Adams et al, 1992; Cleton-Jansen et al, 1995), have been screened for both LOH and mutations.

### MATERIALS AND METHODS

### Tissue samples

Tissues from 47 prostate cancers (16 grade 1, 14 grade 2 and 17 grade 3 carcinomas; grading according to Böcking and Sommerkamp (1980); Helpap et al (1985); age of patients 55–87 years) were obtained from transurethral resections or radical prostatectomy specimens. Thirty-seven tumours were uniform and ten pluriform carcinomas with two or three different malignancy grades. Tissues were formalin-fixed and routinely embedded into paraffin. Representative samples of all malignancy grades of every neoplasm (1–7 areas per tumour) were prepared for LOH analysis by microdissection under microscopic control. In total, 19 grade 1, 45 grade 2 and 33 grade 3 areas were examined.

*Received 28 October 1997*
*Revised 22 May 1998*
*Accepted 2 June 1998*

*Correspondence to:* N Wernert

**Table 1** Allelic losses at 25 chromosomal loci in 97 areas of prostate carcinomas

| | | Samples with LOH/informative samples | | | | | |
|---|---|---|---|---|---|---|---|
| Marker | Chromosomal location | All tumour areas (%) | Grade 1 | Grade 2 | Grade 3 | Frequency of informative samples % (*n*/97) | |
| D7S522 | 7q31.1–q31.2 | 1 (1.6) | 0/11 | 1/28 | 0/22 | 62.8 | (61) |
| D7S523 | 7q31 | 2 (2.6) | 0/14 | 2/34 | 0/32 | 82.5 | (80) |
| D8S87 | 8p21–p12 | 0 (0) | 0/15 | 0/31 | 0/25 | 73.2 | (71) |
| D8S264 | 8p21–pter | 1 (1.3) | 0/16 | 0/38 | 1/26 | 82.5 | (80) |
| D8S265 | 8p23.1 | 3 (5.5) | 0/15 | 2/24 | 1/22 | 63.8 | (61) |
| D11S1392 | 11p13–p12 | 4 (9.1) | 1/14 | 5/32 | 0/26 | 74.2 | (72) |
| D11S904 | 11p15 | 4 (8.5) | 1/14 | 3/24 | 9/14 | 53.6 | (52) |
| D11S488 | 11q23–qter | 4 (7.4) | 0/15 | 1/28 | 3/16 | 60.8 | (59) |
| D12S374 | 12pter–p12 | 5 (7.9) | 1/13 | 3/33 | 0/22 | 70.1 | (68) |
| D12S101 | 12q14 | 7 (11.3) | 1/13 | 4/35 | 2/22 | 72.2 | (70) |
| D12S270 | 12q | 0 (0) | 0/15 | 0/45 | 0/33 | 95.8 | (93) |
| D12S375 | 12q | 6 (7.8) | 0/15 | 3/38 | 3/31 | 86.5 | (84) |
| D13S317 | 13q22–q31 | 5 (6.4) | 0/13 | 1/35 | 6/38 | 88.7 | (86) |
| D13S318 | 13q14.1–q14.3 | 11 (23.9) | 1/15 | 2/19 | 8/18 | 53.6 | (52) |
| D13S325 | 13q21–q22 | 7 (12.5) | 0/15 | 1/23 | 6/24 | 63.9 | (62) |
| D16S398 | 16q22.1 | 5 (7.1) | 0/15 | 2/34 | 3/26 | 78.4 | (76) |
| D16S539 | 16q24–qter | 14 (20.3) | 0/16 | 8/41 | 6/21 | 80.4 | (78) |
| TP53 | 17p13.1 | 11 (23.9) | 0/16 | 7/28 | 4/18 | 63.9 | (62) |
| D17S846 | 17q21 | 5 (9.4) | 0/12 | 3/28 | 2/21 | 62.8 | (61) |
| D17S855 | 17q21 | 0 (0) | 0/15 | 0/37 | 0/30 | 84.5 | (82) |
| D17S250 | 17q11–q12 | 8 (10.8) | 0/16 | 4/35 | 4/29 | 82.5 | (80) |
| D18S549 | 18q | 1 (1.6) | 1/16 | 0/28 | 0/23 | 69.1 | (67) |
| D18S543 | 18q | 0 (0) | 0/14 | 0/30 | 0/17 | 62.8 | (61) |
| D18S541 | 18q21.1–q21.3 | 12 (22.2) | 0/12 | 9/24 | 3/24 | 61.9 | (60) |
| D22S684 | 22q12 | 8 (12.7) | 0/13 | 4/34 | 4/26 | 75.3 | (73) |

### DNA extraction

DNA was extracted from selected tumour areas and normal prostate control tissues after routine deparaffination and proteinase K digestion for 12 h using the QIAamp tissue kit (Qiagen, Hilden, Germany).

### DNA amplification and LOH analysis

Twenty-five different loci on nine chromosomal arms were evaluated for loss of heterozygosity (LOH) by polymerase chain reaction (PCR) amplification of locus-specific polymorphic microsatellite DNA using the following oligonucleotide primers (purchased from Research Genetics, Huntsville, USA): D7S522 (7q31), D7S523 (7q31), D8S264 (8p23), D8S265 (8p23.1), D8S87 (8p12), D11S1392 (11p13), D11S904 (11p14–p13), D11S488 (11q24.1–q25), D12S374 (12pter–p12), D12S101 (12q14), D12S270 (12q), D12S375 (12q), D13S317 (13q22), D13S325 (13q14.1–14.2), D13S318 (13q14.3–q21.1), D16S398 (16q22.1), D16S539 (16q23.1–qter), *TP53* (17p13.1), D17S846 (17q21), D17S855 (17q21), D17S250 (17q11.2–q12), D18S549 (18q), D18S543 (18q), D18S541 (18q21.1–21.3.1) and D22S684 (22q12).

PCR was performed in a final volume of 10 μl containing 10 ng of template DNA, 50 mM potassium chloride, 10 mM tris-HCl, pH 8.3, 200 mM of each dNTP, 0.1% gelatin and 10 pmol of each primer. 0.25 units of *Taq*-DNA polymerase (Gibco BRL) were used. Magnesium chloride concentrations ranged from 1.5 to 2.5 mM, depending upon primer pairs. PCR reactions were carried out on a Biometra UNO-thermocycler. PCR mix in 0.5 ml tubes was overlaid with paraffin oil. For PCR, initial denaturation at 94°C for 3 min was followed by 30 cycles (94°C, 30 s; 52–61°C, 40 s; 72°C, 60 s) and a final elongation step of 10 min at 72°C.

### Gel electrophoresis

PCR products were diluted 1.5:1 in loading buffer (formamide, bromophenol blue and xylene–cyanol) and denatured at 95°C for 5 min. Twelve microlitres of this mixture were run on an 8% polyacrylamide urea sequencing gel at 70 W for 2.5 h in tris-borate buffer. Amplification products were detected by a silverstaining method developed for sequencing gels (von Deimling et al, 1993; Bender et al, 1994).

### Mutational analysis of *TP53* and *BBC1* genes by SSCP and DNA sequencing

For single-strand conformational polymorphism (SSCP) analysis, exons 5–8 of the *TP53* gene and the two exons of the *BBC1* gene were amplified by PCR (magnesium chloride concentration 1.5 mM; 35 cycles: 94°C, 30 s; 60–61°C, 60 s; 72°C, 60 s). Gel electrophoresis was carried out on non-denaturing polyacrylamide gels (6% or 14%, acrylamide:bis-acrylamide 1:30 with glycerol or 1:99 without glycerol, running time 16 h at 8 W at room temperature). Single strands were detected by silverstaining (see above).

Shifted SSCP bands were excised and reamplified by PCR using conditions described above. PCR products were purified with QIAquick PCR Purification Kit (Quiagen). For cycle sequencing, 1 pmol $\mu l^{-1}$ sense or antisense-primer (1.6 μl), 2 μl DNA sequencing kit (Dye Terminator Cycle Sequencing Ready Reaction Mix; Perkin Elmer) and 10–30 ng template DNA (2–3 μl) were

*© Cancer Research Campaign 1999*

**Table 2** Mutations within exons 5–8 of *TP53* in 47 prostate carcinomas

| **Exon *TP53*** | **Mutated codon** | **Nucleotide sequence change** | **Amino acid change** | **Patient number and tumour area (from top to bottom) of Figure 3 (bold numbers indicate LOH at *TP53*)** |
|---|---|---|---|---|
| Exon 5 | 134 | TTT ⇒ GTT | Phe ⇒ Val | 33 (3), 15, **42 (1), 42 (2)**, 38 (2) |
| Exon 5 | 144 | CAG ⇒ CTAG | Insertion T | 24(2) |
| Exon 5 | 165 | CAG ⇒ CAT | Gln ⇒ His | 39 (2) |
| Exon 5 | 177 | CCC ⇒ TCC | Pro ⇒ Ser | 12 |
| Exon 5 | 184 | GAT ⇒ AAT | Asp ⇒ Asn | 39 (1) |
| Exon 6 | 190 | CCT ⇒ CGT | Pro ⇒ Arg | 1 |
| Exon 6 | 193 | CAT ⇒ AAT | His ⇒ Asn | 1 |
| Exon 6 | 200 | AAT ⇒ AAA | Asn ⇒ Lys | **19, 23 (2)** |
| Exon 6 | 200 | AAT ⇒ GAA | Asn ⇒ Asp | **19** |
| Exon 6 | 218 | GTG ⇒ GCG | Val ⇒ Ala | **19** |
| Exon 7 | 239 | AAC ⇒ ATC | Asn ⇒ Ile | 7 |
| Exon 7 | 243 | ATG ⇒ ATA | Met ⇒ Ile | 4 |
| Exon 7 | 249 | AGG ⇒ AAG | Arg ⇒ Lys | 3 |
| Exon 8 | 296 | CAC ⇒ CGC | His ⇒ Arg | 3 |
| Exon 6 | 197 | GTG ⇒ GTA | Silent mutation (Val) | **19** |
| Exon 6 | 213 | CGA ⇒ CGG | Silent mutation (Arg) | **19**, 34 (1) |
| Exon 8 | 275 | TGT ⇒ TGC | Silent mutation (Cys) | 4 |

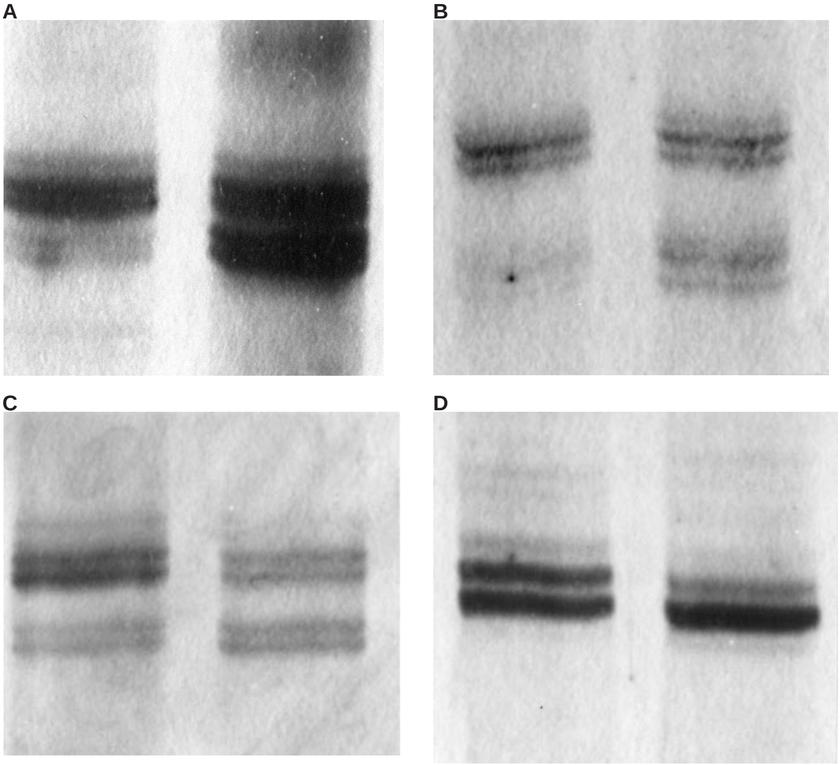

**Figure 1** Examples of allelic losses within four prostate carcinomas. The left (**A**–**C**) or right lanes (**D**) show allelic losses of the lower (**A**–**C**) or the upper allele. The remaining bands are due to fibromuscular stromal cells always present between carcinoma formations. No losses are evident in the normal control DNA (other lanes). (**A**) LOH at D11S904 (11p13) in a grade 2 tumour area (patient 30 of Figure 3, area 1); (**B**) grade 3 carcinoma (patient 36, LOH at D12S101, 12q14); (**C**) grade 3 carcinoma (patient 38, D16S398, 16q22.1); (**D**) grade 3 carcinoma (patient 34, D13S317, 13q22)

used in a final volume of 10 μl (PCR conditions: 96°C, 10 s; 50°C, 5 s; and 60°C, 4 min; 25 cycles). Reaction products were ethanol precipitated, mixed with 4 μl of loading buffer (formamide/EDTA) and denatured for 10 min at 95°C. Products were then electrophoresed through 6% denaturing acrylamide gels using an automatic sequencer (ABI Prism genetic analyser 373, Perkin Elmer). All 97 tumour areas and normal prostate control tissues were examined by SSCP and, in case of shifts, by direct sequencing.

### Evaluation of LOH and statistical analysis

Allelic losses were evaluated by visually comparing alleles of normal DNA with those of tumour DNA. Calculation of fractional allele loss (FAL) was carried out by dividing the number of chromosomal arms with LOH by the total number of informative arms. The *H*-test of Kruskal–Wallis was used to test for statistical differences.

© Cancer Research Campaign 1999 

**Table 3** Mutations within *BBC1* in 47 prostate carcinomas

| Mutated codon | Nucleotide sequence change | Amino acid change | Patient number and tumour area (from top to bottom) of Figure 3 (bold numbers indicate LOH near *BBC1*) |
|---|---|---|---|
| 94 | AGC ⇒ AAT | Ser ⇒ Asn | 24 (1) |
| 129 | ACC ⇒ GCG | Thr ⇒ Ala | 24(1), 46, 29(2), 21(1) |
| 226 | AAG ⇒ AAT | Lys ⇒ Asn | 41(2) |
| 240 | CAG ⇒ CGT | Gln ⇒ Arg | 41 (2) |
| 279 | AAG ⇒ CAG | Lys ⇒ Gln | 21 (2) |
| 290 | AGC ⇒ AAC | Ser ⇒ Asn | **40 (2)** |

### RESULTS

### LOH rates at the 25 chromosomal loci within all tumour samples

The frequency of allelic losses at all 25 chromosomal loci within the 97 tumour areas is given in column 3 of Table 1. No losses at all were found at loci D8S87 (8p21–p12), D12S270 (12q), D17S855 (17q21, within *BRCA1*) and at D18S543 (18q, DCC-region). Figure 1 A–D shows examples of allelic losses. No microsatellite instabilities were observed in the present series of tumours.

### The overall LOH frequency and the fractional allele loss (FAL) are related to malignancy grade

When calculating the mean frequency of allelic losses for all chromosomal markers for the three malignancy grades, losses were found in only 2.2% of grade 1 areas (s.d., 3.4%; minimum, 0%; maximum, 9.1%; median, 0%), but in 9.4% of grade 2 (s.d., 8.3%; minimum, 0%, maximum, 29.4%; median, 5.9%) and in 8.3% of grade 3 areas (s.d., 6.3%; minimum, 0%; maximum, 23.5%; median, 7.8%). The difference between grade 1 on the one hand and grades 2 and 3 on the other was statistically significant at $P = 0.007$.

A similar tendency was found for the mean fractional allele loss (FAL): 0.043 for grade 1 areas (s.d., 0.06; minimum, 0; maximum, 0.12; median, 0), 0.2 for grade 2 areas (s.d., 0.18; minimum, 0; maximum, 0.57; median, 0.12) and 0.23 for grade 3 areas (s.d., 0.18; minimum, 0; maximum, 0.57; median, 0.12). Again the difference between grade 1 and grades 2 and 3 was statistically significant at $P = 0.0004$. There also was a remarkable difference in the number of chromosomal loci affected by LOH between grade 1 on the one hand and grades 2 and 3 on the other. Only six loci were

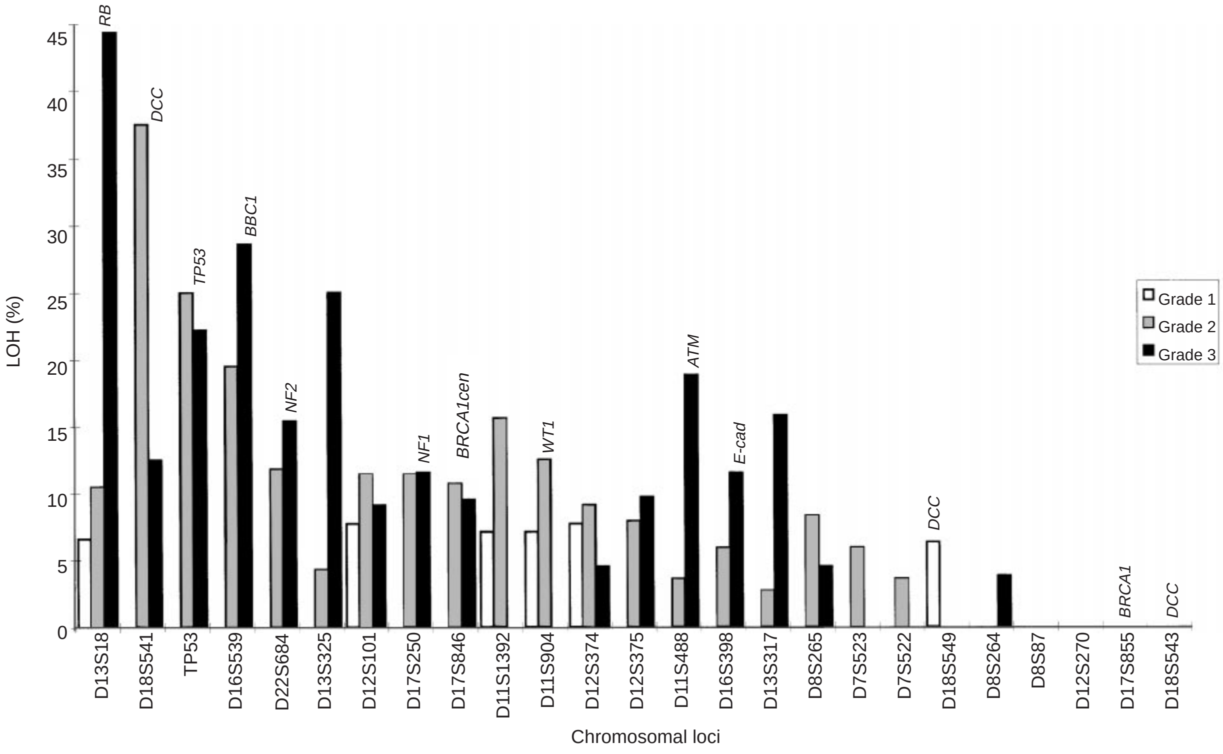

**Figure 2** LOH at individual chromosomal loci in different malignancy grades of 47 prostate carcinomas (97 areas). *ATM*, mutated in ataxia telangiectasia gene; *BBC1*, breast basic conserved gene 1; *BRCA1*, breast carcinoma-associated gene 1; *DCC*, deleted in colon carcinoma; *E-cad*, E-cadherin gene; *NF1*, neurofibromatosis 1 gene; *NF2*, neurofibromatosis 2 gene; *RB*, retinoblastoma gene; *WT1*, Wilms' tumour 1 gene

 © Cancer Research Campaign 1999

affected in grade 1 compared with 19 in grade 2 and 16 in grade 3 areas (see also columns 4–6 of Table 1).

### LOH rates at several loci differ in the three malignancy grades

Considerable differences in LOH frequency between the three histological grades were found at several loci (columns 4–6 of Table 1 and Figure 2). In grade 2 and 3 areas, loss rates between 25% and 44.4% were seen for markers near *RB, DCC, BBC1, TP53* and at *D13S325* (13q21–22). These markers showed losses in only 0–6.6% (D13S318 near *RB*) in grade 1 areas.

### Intra- and intertumoral heterogeneity of LOH

Figure 3 is an overview of the LOH typing at the 25 loci within all 97 tumour areas. In 24 of the 47 tumours (tumours 20–43), 2–7 different areas have been separately evaluated for LOH at all loci. Four of these 24 tumours (tumors 20, 21, 26 and 39) showed no losses at all. Fourteen of the 20 resting cancers (tumours 23, 24, 27–33, 35–38 and 42) presented losses of the same allele at 1–4 loci within two or three different areas. In 5 of these 14 tumours (tumours 29, 31, 33, 35 and 36), these areas were of different grades.

It is also evident from Figure 3 that each individual cancer has its own pattern of LOH. No identical patterns were seen among the 47 tumours.

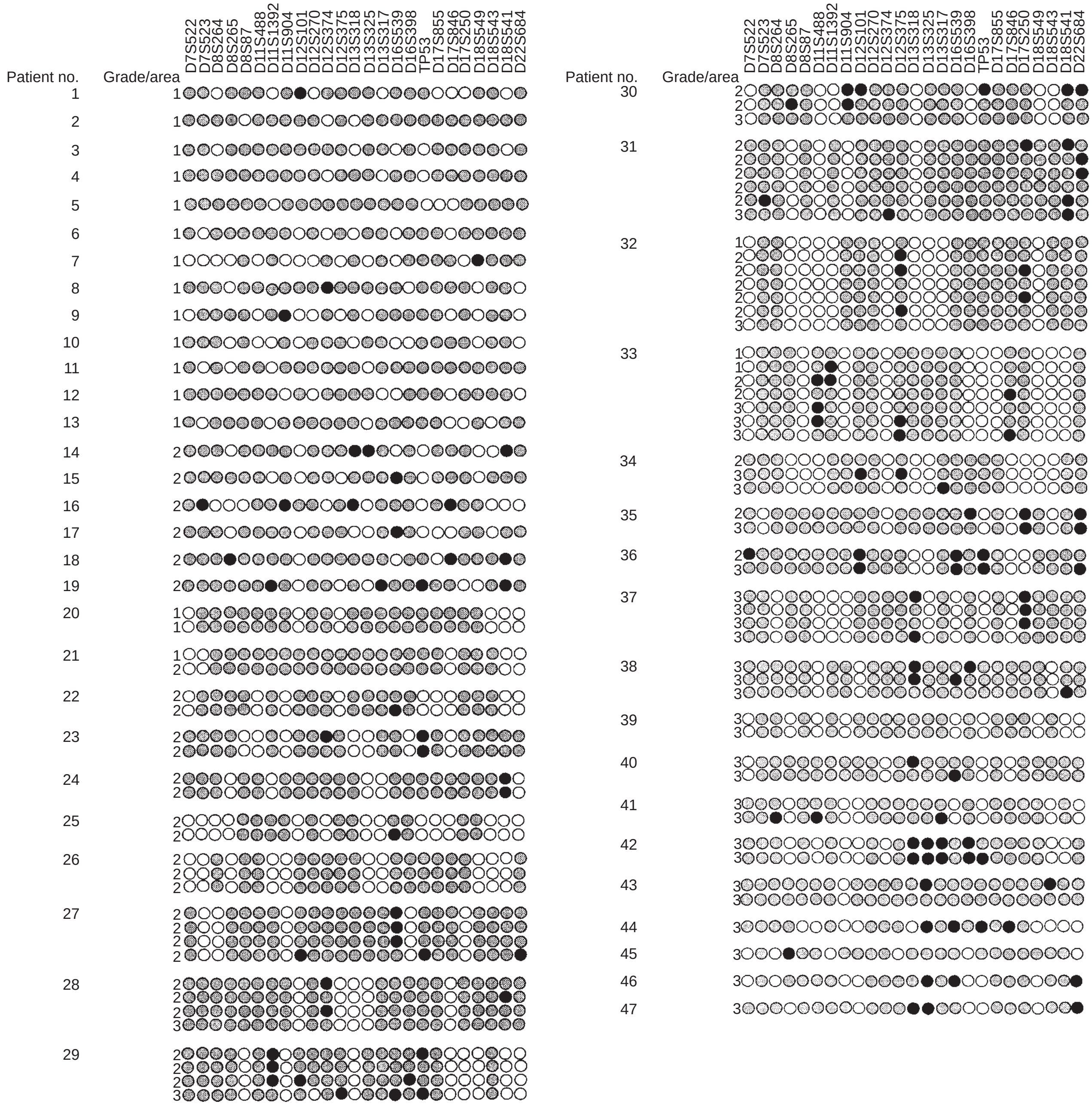

**Figure 3** Summary of LOH typing at 25 chromosomal loci within 47 prostate carcinomas (97 tumour areas). LOH is indicated by black circles and heterozygosity without allelic losses by grey circles. White circles represent non-informative areas

### Mutations of *TP53* and *BBC1* genes

#### *TP53*

Using SSCP analysis and direct sequencing, 18 mutations of exons 5–8 of *TP53* were found within the 97 tumour areas (Table 2), which concerned 14 tumours. Twelve codons were affected by missense mutations, three by silent mutations and one by a T-insertion leading to a stop codon at position 144 (Table 2). Only two carcinomas (tumour 39 and 42) had *TP53* mutations within two different areas (grade 3). These mutations were identical in tumour 42 (codon 134) and different in tumour 39 (codon 165 and 184). Mutations were present in only 4 of the 11 samples with LOH at *TP53* (36%) (Table 2). No correlation was found between tumour grade and mutation frequency.

#### *BBC1*

Mutations within the *BBC1* gene were found in 7 of the 97 samples (six tumours; Table 3). They affected six codons (Table 3). Only carcinoma 21 had two mutations in two areas (grade 1 and 2) which were different (codon 129 and codon 279). In only 1 of the 14 probes with LOH close to *BBC1* was a mutation at codon 290 found (tumour 40, area 2, Table 3). Again, no correlation was evident between tumour grade and mutation frequency.

### DISCUSSION

It is well known that life expectancy of prostate cancer patients is strongly related to tumour grade. Although survival rates are not affected by grade 1 tumours, grades 2 and 3 significantly worsen prognosis (Hanash et al, 1972; Böcking et al, 1982; Dhom, 1991). We wanted to know whether this augmentation of malignant potential goes along with an increase in LOH frequency. We actually found a significant tendency towards both an increase of the overall LOH frequency and the number of affected chromosomal loci with malignancy, when comparing grade 1 areas with grades 2 and 3. This tendency probably reflects an augmenting genetic instability which leads to LOH by mechanisms such as chromosomal losses or interstitial deletions. It is not clear why we found no significant differences between grades 2 and 3 in this series of tumours because microdissection was carried out under microscopic visual control. It can, however, not be excluded that chromosomal loci not examined in the present study differ in their LOH rates between these two grades.

A significant proportion of prostate carcinomas consists of several histological patterns differing in morphology and malignant potential. Whether these patterns are of clonal or multifocal origin is still unclear. LOH typing is a suitable method to study clonality, and special stress was laid upon this point. In 24 of the 47 tumours, 2–7 areas have been systematically analysed for LOH at all 25 chromosomal loci. Four of these tumours had no allelic losses at all. Thirteen of the 20 remaining cancers showed losses at the same allele at 1–4 loci within two or three different areas. In five tumours, these areas were even of different grades. These findings show that there is at least some degree of clonality in prostate carcinoma.

Mutational analyses of *TP53* and *BBC1* did not contribute essentially to the question of clonality in the present series of tumours because only two of them had *TP53* mutations and only one had *BBC1* mutations in two different areas. Mutations were identical in carcinoma 42 (*TP53*), but different in tumours 39 (*TP53*) and 21 (*BBC1*). The findings of two other studies of *TP53* mutations are rather in favour of a multifocal origin of prostate carcinoma (Konishi et al, 1995; Mirchandani et al, 1995).

We found *TP53* mutations in 27% of samples with LOH, which may suggest a certain importance of this TSG in prostate cancer. All mutations concerned the DNA-binding domain encoding region. Those at codons 165, 184, 193, 200, 218, 239, 243 and 296 have not yet been reported (Bookstein et al, 1993; Navone et al, 1993; Chi et al, 1994). Mutations at codons 200 and 243 are known not to affect the DNA-binding properties of the p53 protein (Lin et al, 1994).

The finding of LOH at 16q24–qter (D16S539) near *BBC1* or D16S444E and the demonstration of mutations within this gene in prostate cancers are novel. *BBC1* is a recent candidate tumour-suppressor gene of breast cancers which express it less strongly than benign fibroadenomas (Cleton-Jansen et al, 1995). Homologues have been identified in a wide range of species (Adams et al, 1992; Helps et al, 1995), but the function of the protein is still unknown. The fact that only 7% of prostate cancer samples of this series with LOH near *BBC1* also had mutations is nevertheless not in favour of an important role of this gene in prostate cancer.

Some of the investigated chromosomal loci near *RB, DCC, TP53* and at D13S325 (13q21–22) were found to be more often affected by LOH in grades 2 and 3 compared with grade 1. TSGs close to these sites might, therefore, be important for tumour progression. Fitting in with this view, a suppression of tumorigenicity of prostate cancer cell lines DU-145, TSU and PC-3 which contain mutated *Rb* or *TP53* genes has been achieved upon introduction of the normal genes (Bookstein et al, 1990; Isaacs and Carter, 1991).

A comparison of the 47 carcinomas finally makes evident that each tumour actually has its own pattern of allelic losses when evaluating all 25 loci. Although the LOH typing carried out in this study is far from complete, it is nevertheless tempting to speculate that different combinations of genetic events could result in similar malignant phenotypes of prostate carcinoma, as has also been suggested for other tumours (for review see Macdonald and Ford, 1997).

### ACKNOWLEDGEMENTS

We thank E Bierhoff for his help in sampling the cases. This work was supported by the BONFOR programme of the Faculty of Medicine of the University of Bonn (Germany).

### REFERENCES

Adams SM, Helps NR, Sharp MGF, Brammar WJ, Walker RA and Varley JM (1992) Isolation and characterization of a novel gene with differential expression in benign and malignant human breast tumors. *Hum Mol Genet* **1**: 91–96

Bender B, Wiestler OD and von Deimling A (1994) A device for processing large acrylamide gels. *Biotechniques* **16**: 204–206

Böcking A and Sommerkamp H (1980) Histologisches Malignitätsgrading des Prostatakarzinoms. *Ver Dtsch Ges Urol* **32**: 63–65

Böcking A, Kiehn J and Heinzel-Wach M (1982) Combined histologic grading of prostatic carcinoma. *Cancer* **50**: 288–294

Bookstein R (1994) Tumor suppressor genes in prostatic oncogenesis. *J Cell Biochem* (suppl.) **19**: 217–223

Bookstein R, Shew J, Chen P, Scully P and Lee WH (1990) Suppression of tumorigenicity of human prostate carcinoma cells by replacing a mutated Rb gene. *Science* **247**: 712–715

Bookstein R, MacGrogan D, Hilsenbeck SG, Sharkey F and Allred DC (1993) p53 is mutated in a subset of advanced-stage prostate cancers. *Cancer Res* **53**: 3369–3373

 © Cancer Research Campaign 1999

Buttyan R, Sawczuk IS, Benson MC, Siegal JD and Olsson CA (1987) Enhanced expression of the c-*myc* protooncogene in high-grade human prostate cancers. *Prostate* **11**: 327–337

Chi SG, de Vere-White RW, Meyers FJ, Siders DB, Lee F and Gumerlock PH (1994) p53 in prostate cancer: frequent expressed transition mutations. *J Natl Cancer Inst* **86**: 926–933

Cleton-Jansen AM, Moerland HW, Callen DF, Dogget NA, Devilee P and Cornelisse CJ (1995) Mapping of the breast basic conserved gene (D16S444E) to human chromosome band 16q24.3. *Cytogenet Cell Genet* **68**: 49–51

Cunningham JM, Shan A, Wick MJ, McDonnell SK, Schaid DJ, Tester DJ, Qian J, Takahashi S, Jenkins RB, Bostwick DG and Thibodeau SN (1996) Allelic imbalance and microsatellite instability in prostatic adenocarcinoma. *Cancer Res* **56**: 4475–4482

Dhom G (1991) Pathologie des männlichen Genitale, Tumoren der Prostata. *Spezielle Pathologische Anatomie* **Band 21**: 525–642

Fleming WH, Hamel A, MacDonald R, Ramsey E, Pettigrew NM, Johnston B, Dodd JG and Matusik RJ (1986) Expression of the c-*myc* protooncogene in human prostatic carcinoma and benign prostatic hyperplasia. *Cancer Res* **46**: 1535–1538

Gao X, Honn KV, Grignon D, Sakr W and Chen Y-Q (1993) Frequent loss of expression and loss of heterozygosity of the putative tumor suppressor gene *DCC* in prostate carcinomas. *Cancer Res* **53**: 2723–2727

Gao X, Porter AT and Honn KV (1995*a*) Tumor suppressor genes and their involvement in human prostate cancer: Review *CMB* **2**: 475–498

Gao X, Zacharek A, Grignon D, Liu H, Skr W, Porter AT, Chen YQ and Honn KV (1995*b*) High frequency of loss of expression and allelic deletion of the *APC* and *MCC* genes in human prostate cancer. *Int J Oncol* **6**: 111–117

Gao X, Zacharek A, Salkowski A, Grignon DJ, Sakr W, Porter AT and Honn KV (1995*c*) Loss of heterozygosity of the *BRCA1* and other loci on chromosome 17q in human prostate cancer. *Cancer Res* **55**: 1002–1005

Gumerlock PH, Poonamallee UR, Mayers FJ and deVere White RW (1991) Activated ras alleles in human carcinoma of the prostate are rare. *Cancer Res* **51**: 1632–1637

Hanash KA, Utz DC, Look EN and Taylor WF (1972) Cancer of the prostate: a 15 year follow-up. *J Urol* **107**: 450–453

Helpap B, Böcking A, Dhom G, Faul P, Kastendieck H, Leistenschneider W and Müller HA (1985) Klassifikation, histologisches und cytologisches Grading sowie Regressionsgrading des Prostatakarzinoms. *Pathologe* **6**: 3–7

Helps NR, Adams SM, Brammar WJ and Varley JM (1995) The *Drosophila melanogaster* homologue of the human *BBC1* gene is highly expressed during embryogenesis. *Gene* **162**: 245–248

Isaacs WB (1995) Molecular genetics of prostate cancer. *Cancer Surveys* **25**: 357–379

Isaacs WB and Carter BS (1991) Genetic changes associated with prostate cancer in humans. *Cancer Surveys* **11**: 15–53

Konishi N, Hiasa Y, Matsuda H, Tao M, Tsuzuki T, Hayashi I, Kitahori Y, Shiraishi T, Yatani R and Shimazaki J (1995) Intratumor cellular heterogeneity and alterations in ras oncogene and *p53* tumor suppressor gene in human prostate carcinoma. *Am J Pathol* **147**: 1112–1122

Kuhn EJ, Kurnot A, Sesterhenn A, Chan EH and Moul JW (1993) Expression of the c-erbB-2 (HER-2/neu) oncoprotein in human prostatic carcinoma. *J Urol* **150**: 1427–1433

Latil A, Fournier G, Cussenot O and Lidereau R (1996) Differential chromosome allelic imbalance in the progression of human prostate cancer. *J Urol* **156**: 2079–2083

Lin J, Wu X, Chen J, Chang A and Levine AJ (1994) Functions of the p53 protein in growth regulation and tumor suppression. *Cold Spring Harbor Symp Quant Biol* **59**: 215–223

Macdonald F and Ford CHJ (1997) *Molecular Biology of Cancer*. BIOS Scientific Publishers: Oxford

Macoska JA, Trybus TM, Benson PD, Sakr WA, Grignon DJ, Wojno KD, Pietruk T and Powell IJ (1995) Evidence for three tumor suppressor gene loci on chromosome 8p in human prostate cancer. *Cancer Res* **55**: 5390–5395

Mirchandani D, Zheng J, Miller GJ, Ghosh AK, Shibata DK, Cote RJ and Roy-Burman P (1995) Heterogeneity in intratumor distribution of p53 mutations in human prostate cancer. *Am J Pathol* **147**: 92–101

Moul JW, Friedrichs PA, Lance RS, Theune SM and Chang E (1992) Infrequent RAS oncogene mutations in human prostate cancer. *Prostate* **20**: 327–338

Navone NM, Troncoso P, Pisters LL, Goodrow TL, Palmer JL, Nichols WW, von Eschenbach, AC and Conti CJ (1993) p53 protein accumulation and gene mutation in the progression of human prostate carcinoma. *J Natl Cancer Inst* **85**: 1657–1669

Peehl DM (1993) Oncogenes in prostate cancer: an up-date. *Cancer* (suppl.) **71**: 1159–1164

Sadasivan R, Morgan R, Jennings S, Austenfeld M, van Veldhuizen P, Stephens R and Noble M (1993) Overexpression of HER-2/NEU may be an indicator of poor prognosis in prostate cancer. *J Urol* **150**: 126–131

Sandberg AA (1992) Cytogenetic and molecular genetic aspects of human prostate cancer: primary and metastatic. In Karr JP and Yamanoka H (eds) *Prostate Cancer and Bone Metastasis* **324**: 45–75

Trapman J, Sleddens HFBM, van der Weiden MM, Dinjens WNM, Konig JJ, Schroder FH, Faber PW and Bosman FT (1994) Loss of heterozygosity of chromosome 8 microsatellite loci implicates a candidate tumor suppressor gene between the loci D8S87 and D8S133 in human prostate cancer. *Cancer Res* **54**: 6061–6064

Umbas R, Schalken JA, Aalders TW, Carter BS, Karthaus HFM, Schaafsma HE, Debruyne FMJ and Isaacs WB (1992) Expression of the cellular adhesions molecule E-cadherin is reduced or absent in high-grade prostate cancer. *Cancer Res* **52**: 5104–5109

Viola MV, Fromowitz F, Oravez S, Deb S, Finkel G, Lundy J, Hand P, Thor A and Schlom J (1986) Expression of ras oncogene p21 in prostate cancer. *N Engl J Med* **314**: 133–137

Vocke CD, Pozzatti RO, Bostwick DG, Florence CD, Jennings SB, Strup SE, Duray PH, Liotta LA, Emmert-Buck R and Linehan WM (1996) Analysis of 99 microdissected prostate carcinomas reveals a high frequency of allelic loss on chromosome 8p12–21. *Cancer Res* **56**: 2411–2416

von Deimling A, Bender B, Louis DN and Wiestler OD (1993) A rapid and non-radioactive PCR based assay for the detection of allelic loss in human gliomas. *Neuropathol Appl Neurobiol* **19**: 524–529

Wales MM, Biel MA, el Deiry W, Nelkin BD, Issa JP, Cavenee WK, Kuerbitz SJ and Baylin SB (1995) p53 activates expression of HIC-1, a new candidate tumour suppressor gene on 17p13.3. *Nature Med* **1**: 570–577

Zenklusen JC, Thompson JC, Troncoso P, Kagan J and Cont CJ (1994) Loss of heterozygosity in human primary prostate carcinomas: a possible tumor suppressor gene at 7q31.1. *Cancer Res* **54**: 6370–6373

© Cancer Research Campaign 1999
